# Health System Resilience: Withstanding Shocks and Maintaining Progress

**DOI:** 10.9745/GHSP-D-22-00076

**Published:** 2022-09-15

**Authors:** Agnes Binagwaho, Dennis Hirwe, Kedest Mathewos

**Affiliations:** a University of Global Health Equity, Kigali, Rwanda.

## Abstract

Building a resilient system capable of responding to health threats while maintaining essential health service delivery requires health systems to embed implementation research capacity to create the knowledge needed, ensure the affordability and accessibility of health care services at all levels of delivery, and provide the range of services needed to meet the population’s needs.

## Key Messages

- As evidenced by the 2014–2016 Ebola outbreak and the current coronavirus disease (COVID-19) pandemic, the lack of resilient health systems results in an ineffective and delayed response to emergency health threats and disruptions to the delivery of essential health services.
- Embedding implementation research within health systems enables health care workers and policy makers to rapidly identify facilitating and challenging contextual factors as well as to devise and adapt strategies to respond to emerging obstacles.
- Ensuring accessibility and affordability of health care and building a health system capable of providing a range of services starting at the primary care level between crises is crucial to maintaining progress in health outcomes during a health threat.
- Implementation science can play a critical role in sharing transferable lessons on how strategies and programs can be implemented to build and maintain resilient health systems.

## INTRODUCTION

The call for resilient health systems often increases during and shortly after every emergency health crisis. This was the case after the 2014–2016 Ebola virus disease (EVD) outbreak, and it is of even greater scale now during the coronavirus disease (COVID-19) pandemic.^1^ The high number of deaths caused directly by the Ebola virus in the 3 most hit West African countries—Guinea, Liberia, and Sierra Leone—was explained by the lack of efficiency of their health systems. However, the unexpectedly high number of deaths not directly linked to the EVD but instead to the lack of availability of essential health services was explained by its lack of resiliency. These countries were not only unable to rapidly and effectively respond to the new health threat as they were unprepared, but they were also unable to maintain consistent and high-quality provision of routine, essential health services while they were responding to the EVD outbreak. Consequently, they faced major disruptions in health care service delivery and in social programs.^2^ During the COVID-19 pandemic, while the case fatality rate of the coronavirus is significantly lower than that of the Ebola virus,^3^ the rapid global spread of the virus and the duration of the pandemic have negatively affected all countries worldwide. As a result, the indirect consequences on the health system’s capacity to deliver essential health services when facing a public health threat have become more visible, with more than 90% of countries reporting some level of disruption.^4^ Usually such circumstances affect low- and middle-income countries (LMICs) the most. However, this time, because high-income countries (HICs) have been affected as well, the importance of resilient, data-driven health systems that are capable of tackling emerging crises while maintaining core function is at the forefront of global health discussions, now more than ever.

Health system resilience is often defined through frameworks that highlight the characteristics of a health system that demonstrate it is prepared to withstand health shocks while maintaining the delivery of routine health services provided to the population. A well-cited framework in this field is that of Kruk et al. developed after the 2014–2016 Ebola crisis. They define health system resilience as^2^:

*the capacity of health actors, institutions, and populations to prepare for and effectively respond to crises; maintain core functions when a crisis hits; and, informed by lessons learned during the crisis, reorganize if conditions require it.*

Health system resilience is often defined through frameworks that highlight the characteristics of a health system that is prepared to withstand health shocks while maintaining the delivery of routine health services.

Kruk et al. identified various preconditions that enable a resilient health system, including awareness of the severity of the health threat, a clear understanding of all actors’ roles at the local and global levels, a conducive legal and policy environment, a strong and committed health workforce, and social capital. Drawing from various resilience frameworks in other fields, they identified 5 characteristics of resilient health systems: have awareness of existing gaps, strengths, and opportunities to address challenges; be diverse enough to address a wide range of health needs; be self-regulating in their capacity to isolate the threat and maintain core function; be integrated to bring together diverse stakeholders and ideas; and be adaptive enough to transform function as necessary.^2^ These characteristics of a resilient health system need to be integrated into all levels of the health care system, starting at the primary care level, which has the largest exposure to communities.

## BUILDING RESILIENT HEALTH SYSTEMS WITH EMBEDDED IMPLEMENTATION RESEARCH

Ensuring that delivery system actors at policy and implementation levels have the tools, training, and opportunities to learn is central to building a resilient health system. This is because continuous inquiry, knowledge generation, use of evidence, and adaption to local context are intrinsic to the 5 characteristics of resilience. A critical component of this is developing country-level capacity to perform implementation research (IR) which is defined as^5^:

*the scientific study of the use of strategies to adopt and integrate evidence-based health interventions into clinical and community settings to improve individual outcomes and benefit population health.*

Often, the cause of poor health outcomes is not the lack of knowledge about quality medical interventions but rather the inability of our health systems to implement them effectively and equitably. This lack of implementation know-how is a significant contributor to the gap between the discovery of evidence-based interventions and their widespread availability throughout health systems.^6^ IR helps identify the contextual factors that either facilitate or hinder progress and the strategies that can be used to implement the interventions to get the desirable outcomes. Thus, in the context of health crises such as COVID-19, adopting an IR approach allows health systems to rapidly identify factors that can interrupt health service delivery, the strategies that could be used in response, and those that will facilitate the delivery of essential health services. Training health care workers, health system managers, policy makers, in-country researchers, and other relevant stakeholders in IR either in designing and conducting research or in research utilization is thus critical to building a flexible health system. Linking back to the resilience framework previously mentioned, this culture of learning and continuous improvement enables the health system to become more aware of any challenges and the locally relevant and effective strategies to overcome them. It also enables the health system to become more adaptive to arising circumstances.

The lack of implementation know-how is a significant contributor to the gap between the discovery of evidence-based interventions and their widespread availability throughout health systems.

Equipping the relevant stakeholders with the capacity to seek improvement, including through IR, will enable them to continually improve health system delivery and responsiveness. This ability to learn and adapt is especially important at the community level in many countries where part of primary care is managed by community health workers, nurses, and midwives who often do not have high-level credentials or training in research. In addition to the training health workers receive as they begin their tasks, health systems need to create a culture of continuous education to enable the health workforce to identify questions to be answered and understand what strategies work best in their context. This culture of continuous improvement can only be built if health systems invest in research capacity building on formulating and pursuing relevant research questions and have the political will to translate research results into responsive policies and programs. Training also needs to build the capacity of engaged stakeholders to utilize the knowledge emerging from such research. Additionally, such a learning health system needs to be supported by platforms for knowledge sharing within and between countries. Knowledge exchange programs such as the recent International Conference on Public Health in Africa have been initiated to share the knowledge and expertise that exists on the continent.^7^

## BUILDING HEALTH SYSTEM RESILIENCE BY INCREASING AFFORDABILITY AND ACCESSIBILITY TO HEALTH CARE SERVICES

Building health system resilience needs to start at the primary health care (PHC) level, which is the first point of contact for the community to the health system before and during health crises. Strengthening PHC systems could save 60 million lives and increase life expectancy by 3.7 by 2030 in LMICs.^8^ Thus, ensuring consistent implementation outcomes such as affordability and accessibility of holistic services at the primary level is critical to withstand shocks to the health system. IR can be particularly helpful in determining, evaluating, and scaling up the strategies and programs needed to ensure the equitable rollout of evidence-based interventions in a given context.^9^ Studying the implementation of ongoing programs targeted at ensuring accessibility can help policy makers identify example strategies that could be adopted within their context. One such successful program is Rwanda’s Community Based Health Insurance program known as Mutuelle de Sante, which has helped increase and maintain access to PHC, currently covering more than 90% of the population, up from 36% in 2006.^10^ Community-based health insurance programs reduce catastrophic expenditures which put more than 930 million people globally at risk of poverty.^8^ This becomes especially important during health crises that threaten households’ incomes and hinder their ability to seek health services.

Beyond increasing financial accessibility, there is a need to ensure geographic accessibility. IR studies have shown the importance of understanding local contextual factors such as geographic obstacles that impede health care access and the strategies that are effective in addressing them.^11^ Such studies also provide transferable knowledge for countries to adapt and adopt to improve health outcomes. For instance, investing in increasing the number of health centers at the community level has been found to contribute toward geographic equity and the reduction of travel distance to quality health services, especially for a population with limited access to vehicles.^12^ In Rwanda, the decentralization of health facilities aligned to the country’s administrative structure can be an example, with 58,000 community health workers at the village level, 1,700 health posts at the cell level (villages are organized into cells), 500 health centers at the sector level, 42 district hospitals, and 5 national referral hospitals across the country that resulted in a considerable reduction in the average time to access the nearest health facility from 95.1 minutes in 2006 to 49.9 minutes in 2017.^13^ Community health systems are also central to increasing access to PHC and require further investments in the training and incentivization of community health workers. For example, in Africa, Rwanda, Ethiopia, Senegal, Kenya, and Botswana made major investments in community health systems that consequently contributed significantly to the reduction of morbidity and mortality in the countries from 2005 to 2015. The overall burden of disease during this period was reduced by 48% in Rwanda, 39% in Ethiopia, 38% in Kenya, 46% in Botswana, and 36% in Senegal.^14^

IR studies have shown the importance of understanding local contextual factors that impede health care access and the strategies that are effective in addressing them.

Financing the accessibility and affordability of PHC in LMICs can start at the domestic level instead of waiting for international contributions. For instance, the government of Rwanda finances Mutuelle de Sante from the national budget and contributions from other sources, such as through the Rwanda Social Security Board and other subsidies that include 10% of fees collected from road traffic fines, 2.5%–3% of the telecommunication sector’s annual turnover, 50% of pharmaceutical products and medical devices’ registration fees, 100% of the funds collected as medical research fees collected by the Ministry of Health, and 50% of the motor vehicle mechanical inspection fees with 10% of road traffic fines paid to the Rwanda National Police.^15^ The government spends 7.5% of its gross domestic product on health, which is 2.5 percentage points above the suggested expenditure of 5% of gross domestic product needed to achieve universal health coverage.^16^ Such sources of income need to be generated and used to finance PHC systems that contribute to resilience. COVID-19 has shown us that a lack of resilient health systems can lead to the disruption of health services and increase morbidity due to causes other than a direct health threat. It is possible to harness IR frameworks and constructs to promote resilience and understand whether and how strategies promote sustainability of essential health services over time. For example, the Exploration, Preparation, Implementation, and Sustainment framework emphasizes sustainability integrated as a critical part of implementation.^17^ Additionally, sustainability, defined as “the extent to which a newly implemented treatment is maintained or institutionalized within a service setting’s ongoing, stable operations,”^18^ is often an implementation outcome that strategies are evaluated against.

## USING IMPLEMENTATION SCIENCE TO IMPLEMENT BEST PRACTICES THAT ADDRESS EMERGING PUBLIC HEALTH CONCERNS

IR studies can help provide the next steps for epidemiological studies that guide our understanding of disease burden in a country. IR can inform the programs and strategies to be implemented to effectively address new or emerging threats. For instance, in low-income regions such as Africa, we need to increasingly pay special attention to the changing disease burden and prepare a diverse health system to provide the needed new services to respond to the population’s health needs—an important characteristic of a resilient health system. Given the increase in life expectancy in Africa,^19^ and the reduction in infectious disease burden, the new major challenge that needs epidemiological attention are noncommunicable diseases (NCDs) that were responsible for 36.4% of total deaths in sub-Saharan Africa in 2019.^20^ Thus, we need to prepare our health systems to address this rising burden. Using IR to understand what other regions that have undergone this epidemiological transition have done can provide useful lessons for countries currently facing this challenge. Once best practices from other regions are identified, there is a role for IR to guide the adaptation of best practices to the local context as well as their integration and spread throughout health systems. We can take the example of Rwanda, which in its action plan for Prevention and Control of NCDs, has identified the need for an investment of over US$353 million from 2020 to 2025 to improve screening, diagnosis, and treatment of NCDs, as well as to strengthen advocacy and training of health workers, with the ultimate goal of reducing premature mortality from NCDs by 25% in 2025.^21^ The plan advocates for a multisectoral equity-based approach to successfully oversee its successful execution. An implementation science study examining how a low-income country such as Rwanda finances NCD care and prepares its system for care provision can provide helpful lessons for similar countries seeking to reduce NCD burden.

We need to prepare a diverse health system to provide the needed new services to address the population’s health needs in response to the changing disease burden—an important characteristic of a resilient health system.

From Senegal’s experience, the “Better City Better Hearts” initiative, which was initiated through a partnership between the Ministry of Health and Social Action, IntraHealth International, the Novartis Foundation, PATH, local health officials, community-based organizations, and other local stakeholders, aims to address hypertension and improve cardiac health among the population of Dakar. This initiative has trained 667 health workers across 3 districts in Dakar and increased the number of health workers with hypertension knowledge by 22% from 2017 to 2019.^22^ We can also refer to low-resource solutions like the Car Free Day in Rwanda—a twice-monthly initiative in Kigali where cars are banned from main roads to allow citizens to cycle, walk, and jog to designated assembly points from 7 to 11 am.^23^ Leveraging this initiative, the Ministry of Health and the NCD Division of the Rwanda Biomedical Center in 2019 set up tents to provide free NCD screenings after stretching exercises—eye, dental, diabetes, and counseling among others. In 2019, the participation was 20,000, and the activity was extended to other cities.^24^ On average, 7,000 people regularly benefit from these services every year. This screening and early detection initiative can further support the prevention of NCDs—a major disease burden in Africa that as of 2019 accounts for 32% of deaths in sub-Saharan Africa.^25^

There are 2 takeaways here. First, adopting and adapting such lessons from countries that have successfully developed initiatives and strategies to make their health systems more resilient is necessary to make rapid improvements in health outcomes. Secondly, building collaborative networks such as the one in Senegal that enabled the initiative “Better City Better Hearts” is an example of an approach to building an integrated health system—another characteristic of health system resilience. IR studies allow us to look beyond the clinical outcomes of an intervention or policy to the strategies that were used to implement them, the facilitating and challenging contextual factors, and the implementation outcomes.

IR studies allow us to look beyond the clinical outcomes of an intervention or policy to the strategies that were used to implement them, the facilitating and challenging contextual factors, and the implementation outcomes.

## BUILDING HEALTH SYSTEM RESILIENCE PROACTIVELY

It is critical that health system resilience be sought proactively, not during public health threats. The focus of the health system during a crisis such as COVID-19 is responding to the shock through surveillance, public communication, and the development of testing, treatment, vaccination, and other prevention guidelines among other strategies. A resilient health system enables the implementation of such strategies while at the same time facilitating the prevention or mitigation of the disruption of regular functions. To illustrate this point, we can look at the results of a mixed-methods hybrid IR study conducted in Rwanda to understand the maintenance of child health interventions such as vaccinations during the COVID-19 pandemic (unpublished data). Rwanda exhibited remarkable maintenance of these essential health interventions between March and December 2020, with reductions in delivery in the early phases of the pandemic that coincided with strict national lockdowns. The country implemented various strategies such as community-based health care delivery, data use for decision making, communication, and leveraged facilitating contextual factors such as strong coordination and robust supply chain system to address challenges to health care delivery that were either directly related to the spread of the virus or the resulting public health response. While some of the strategies were adapted to various degrees to the emerging context, almost all of them existed before the pandemic and contributed to the creation of a resilient health system. The use of existing strategies and the ability to leverage existing facilitating contextual factors to respond to a new health threat is evidence of resilience at its core (i.e., evidence of a self-regulated health system that is prepared to weather shocks, mitigate challenging contextual factors, and maintain core function).

## CONCLUSION

Health system resilience is a prerequisite to maintaining health security during current and future health threats. Although COVID-19 surprised the entire world, it is not the last pandemic or health threat the world will face. We have already seen that the consequences of climate change impact health systems and population health. Thus, understanding the components of a resilient health system and how to build them, while continuing to provide essential health services is central to continuing to make progress in health outcomes. To understand and use such knowledge, it is critical to embed IR in health care systems not only to improve health service delivery during ordinary times but also to enable the workforce to rapidly adapt health systems to emerging situations, maintain core functions, and accelerate the regional and global learning and uptake from this emerging knowledge. The COVID-19 pandemic has shown that if we are not prepared to identify the health threat and devise strategies to respond to both the direct and indirect challenges to health service delivery, this lack of resilience presents an enormous obstacle to the achievement of universal health coverage and the Sustainable Development Goals.

## Notes

**Peer Reviewed**
